# Dynamics and Diversity of Intrauterine Anaerobic Microbiota in Dairy Cows with Clinical and Subclinical Endometritis

**DOI:** 10.3390/ani13010082

**Published:** 2022-12-26

**Authors:** Panagiotis Ballas, Harald Pothmann, Isabella Pothmann, Marc Drillich, Monika Ehling-Schulz, Karen Wagener

**Affiliations:** 1 Clinical Unit for Herd Health Management in Ruminants, University Clinic for Ruminants, Department for Farm Animals and Veterinary Public Health, University of Veterinary Medicine Vienna, 1210 Vienna, Austria; 2 Functional Microbiology, Institute of Microbiology, Department for Pathobiology, University of Veterinary Medicine Vienna, 1210 Vienna, Austria

**Keywords:** intrauterine infection, anaerobic bacteria, endometritis, cattle, microbial community

## Abstract

**Simple Summary:**

Research on uterine bacterial communities in diseased and healthy animals is crucial for a better understanding of the development of uterine diseases. Therefore, the objective of the study was to characterize the dynamics of anaerobic cultivable microbiota in the uterus of dairy cows. Sampling of 122 dairy cows at six time points after calving resulted in 1858 bacterial isolates. Remarkable shifts were observed in the composition of microbiota throughout the postpartum period, whereas some bacteria were associated with endometritis, and some were frequently detected in healthy animals. This study provides information for the development of novel prevention and therapeutic strategies.

**Abstract:**

The aim of the study was to characterize the dynamics of anaerobic cultivable postpartum microbiota in the uterus of dairy cows. In total, 122 dairy cows were enrolled and sampled on day 0 (day of calving) and on days 3, 9, 15, 21, and 28 postpartum (pp). Samples were cultivated anaerobically and analyzed by MALDI-TOF MS. In total, 1858 isolates were recovered. The most prevalent facultative anaerobic genera were *Trueperella* (27.8%), *Streptococcus* (25.4%), and *Escherichia* (13.1%). The most prevalent obligate anaerobes were *Peptoniphilus* (9.3%), *Bacteroides* (3.3%), and *Clostridium* (2.4%). The microbial communities were highly dynamic and diverse. On the animal level, *Trueperella pyogenes* on day 21 and 28 pp was associated with clinical endometritis, and *E. coli* on day 21 pp was associated with subclinical endometritis. The occurrence of *Streptococcus pluranimalium* on day 28 was related to uterine health. The presence of *T. pyogenes*, *Streptococcus*, and *Peptoniphilus* was significantly associated with an increased risk for purulent vaginal discharge. Primiparous cows showed a higher prevalence of *T. pyogenes*, *Fusobacterium necrophorum*, *Porphyromonas levii*, and *Peptoniphilus* spp. than multiparous cows but were not more susceptible to uterine diseases. This study might provide a suitable basis for future co-cultivation studies to elucidate potential synergistic interactions between microbiota.

## 1. Introduction

Microbial dysbiosis, the animal immune response, and tolerance against pathogens are decisive for the development of uterine diseases, such as metritis, clinical endometritis (CE), and subclinical endometritis (SE) [1]. Research on the uterine bacterial communities in diseased and healthy animals is crucial for the understanding of the pathogenesis of uterine diseases and represents the basis for the development of novel prevention and therapeutic strategies.

Previous notions that the bovine uterus is sterile before parturition are being challenged by recent studies, which indicate that the pregnant and also the virgin uterus harbors its own, unique microbiome [2,3]. Additionally, at the time of insemination, the uterus contains a high diversity of bacteria, which are not related to uterine disease or subfertility [4]. Numerous studies compared the uterine microbiome at the time of CE or SE diagnosis between cows with uterine diseases and healthy cows. The studies were aiming to differentiate between commensal bacteria associated with health and pathogenic bacteria associated with disease. In a recent study, *Trueperella pyogenes* was found as the only bacteria associated with CE, and obligate anaerobes were rarely detected [5]. Obligate anaerobes such as *Bacteroides, Fusobacteria*, *Peptoniphilus*, and *Porphyromonas* have mainly been linked to clinical or puerperal metritis [6,7] but were also found, besides *T. pyogenes*, in high prevalence at the day of CE diagnosis [8]. In contrast to metritis and CE, SE does not seem to be a result of bacterial persistence. No association between SE and bacterial findings was observed on day 21 to 62 [9] or day 20 to 30 [10] postpartum. In addition, Baranski et al. [11] reported only a low correlation between cytological and bacteriological findings in the fourth and sixth week postpartum. Cows with SE, however, showed a greater bacterial growth density on day 3, 9, and 15 postpartum compared to healthy animals. Thus, bacterial invasion before diagnosis appears to be relevant for the development of SE [12]. The complex diversity and dynamics of uterine microbiota suggest that changes in the entire bacterial community are of higher relevance for disease development than single bacterial findings [13].

To gain in-depth insights into disease development and depict dynamics of bacterial communities, repetitive sampling before, at the time of, and after disease diagnosis is necessary [13,14]. Studies on the dynamics of intrauterine aerobic microbiota showed that the first difference in the aerobic bacterial composition between healthy cows and cows with CE and SE were already present around the second week postpartum (pp) [13,15,16]. The early presence of *T. pyogenes* (day 10 to 15 pp) was associated with the development of CE [13] and SE [15]. Furthermore, the presence of *Streptococcus uberis*, i.e., α-hemolytic streptococci, in the first two weeks pp was found to be related to subsequent CE [13] and SE development [15]. 

Detailed information on the dynamics of obligate or facultative anaerobic microbiota in cows with CE or SE is still lacking. To our knowledge, no culture-dependent studies exist on the dynamics of anaerobic microbiota in cows with CE and SE before, during, and after disease diagnosis development, and only a limited number of cultivation-independent studies have examined microbiota compositional changes around disease diagnosis [17,18]. 

Culture-independent studies enable a general overview of the microbiome but do not allow functional host–microbiota studies. Complementary culture-dependent studies, providing the basis for later experimental in vitro studies on microbiota–host interactions, are essential for a better understanding of the pathophysiology of postpartum uterine disease [19,20]. Therefore, the objective of this study was to monitor compositional changes in the intrauterine anaerobic cultivatable microbiota in healthy cows as well as in cows with CE and SE to relate bacterial findings during disease development with the host uterine health status. 

## 2. Materials and Methods

This study has been approved by the institutional ethics committee and the national authority according to § 8 of Law for Animal Experiments (Tierversuchsgesetz-TVG BMWF-68.205/246-II/3b/2010).

### 2.1. Study Farm and Study Animals

The bacterial isolates analyzed in the frame of the current study represent the anaerobic fraction of uterine bacteriological samples collected previously [13]. The initial collection of uterine bacteriological samples was performed between February 2011 and February 2013 at the teaching and research farm of the University of Veterinary Medicine Vienna, VetFarm Kremesberg. Dairy cows (60% Simmental, 15% Holstein Friesian, and 25% Swiss Brown) were housed in free-stall barns with straw-bedded cubicles. The ration consisted of grass silage, maize silage, and hay and was supplemented with minerals and concentrate. In total, 170 calvings were initially enrolled. Animals with cesarean section, vaginal lacerations, and downer cows were not included in the study. Sampling was initiated within 12 h after calving (day 0) and continued on days 3, 9, 15, 21, and 28 pp. A short clinical examination was performed before sampling. Animals with systemic signs of illness and a rectal temperature above 39.5 °C and fetid, red-brown, watery uterine discharge were considered as suffering from metritis, as described previously [21]. Animals with metritis received systemic antibiotic treatment with 1 mg/kg of ceftiofur (Excenel Flow, Zoetis, Berlin, Germany) and were excluded from further sampling. Another 27 cows that received antibiotic treatment for other reasons, e.g., clinical mastitis and orthopedic disorders, were excluded. Six cows were excluded, as sampling was not possible on each time point, and the data set was incomplete. After the aforementioned exclusions, a population of 122 animals remained in the final analysis.

### 2.2. Gynecological Examination and Diagnosis of Clinical and Subclinical Endometritis

In addition to a short physical examination, a gynecological examination was performed on day 21 pp by vaginal inspection and rectal palpation of the uterus. Vaginoscopy was performed with a speculum and a torch as described before [22]. The vaginal discharge was classified according to Sheldon et al. [21]. Cows with clear vaginal discharge were categorized as vaginal discharge score (VDS) 0, discharge with flecks of pus was considered as VDS 1, animals with discharge with <50% pus were considered as VDS 2, and when >50% purulent material was present, cows were classified as VDS 3 according to Sheldon et al. [21]. Diagnosis of SE was performed as previously described [12]. In brief, animals with VDS 0 and <5% PMN in the cytological smear were considered as healthy (HE), animals with VDS 0 and ≥5% PMN were defined as having SE, and animals with VDS 1, 2, or 3 were considered as animals with CE.

### 2.3. Bacteriological Sampling

Sampling was performed by using the cytobrush technique [23] as described before [12,13]. The samples were transferred into sterile phosphate-buffered saline (PBS, Biochrom, Berlin, Germany). Within one hour from the initial sampling, the brushes were rolled onto Columbia III agar supplemented with 5% sheep blood (Beckton Dickinson, Heidelberg, Germany). The plates were incubated anaerobically at 37 °C for 48 h by using gas packs (GenBag Anaerobic, Biomerieux, Marcy-l’Étoile, France). Each colony was streaked out again to obtain a pure culture. Single colonies were prepared and frozen at −80 °C in 20% *v*/*v* glycerol until further analysis. For the matrix-assisted laser desorption ionization–time of flight mass spectrometry (MALDI-TOF MS), the bacterial isolates were revived on Columbia III agar supplemented with 5% sheep blood. Incubation was performed anaerobically at 37 °C for 48 h. Bacterial biomass of single colonies was collected, and protein extraction and MALDI measurements were performed as previously described [24]. 

Spectral analyses were performed by using the MBT Compass Explorer software (ver. 4.1.60, server database: 4.1.60 (PYTH) 28 2016-04-18_11-26-19; Bruker Daltonics, Billerica, MA, USA) provided by the manufacturer. The score values were interpreted according to the manufacturer’s instructions. Score values higher than 2.00 indicated species identification, while values between 1.70 and 2.00 indicated genus-level identification. A score value lower than 1.70 indicated that the bacterial identification was unsuccessful. For bacteria that were not identified by MALDI-TOF MS, a partial amplification of the 16S rRNA gene was performed. Bacteria were grown anaerobically, and then, bacterial genomic DNA was extracted using the MasterPure Gram-positive DNA extraction kit (Epicentre Biotechnologie, Madison, WI, USA) according to the manufacturer’s instructions. Amplification of the 16S rRNA gene was performed with the modified versions of the universal primers 27f and 1492r (forward: AGAGTTTGATCCTGGCTCA, and reverse: CGGCTACCTTGTTACGAC) as described before [24]. Sequencing, editing, and similarity check was done as described before [24]. Bacterial isolates with less than 98.65% 16S rRNA similarity with the closest defined bacterial species were considered as hitherto potentially undescribed bacterial species [25]. 

### 2.4. Statistical Analysis 

All statistical analyses were performed using SPSS (IBM SPSS Statistics for Windows, Version 25. Armonk, NY, USA). Differences in the proportion (animal level) and abundances (isolate level) of bacteriological findings on species and genus level were tested between animals with different health status (HE vs. CE, HE vs. SE, and CE vs. SE) and parity (primiparous vs multiparous) by using the *χ*^2^-test. The Fisher’s exact test was applied when the expected values in one of the cells of the contingency table was <5. A multinomial logistic regression with backward elimination of variates was used to associate bacterial findings (0 = negative; 1 = positive) on certain days with the VDS (reference = HE animals, i.e., VDS 0 with no SE). The bacterial findings (0 = negative; 1 = positive) were also associated with the presence of SE by using a binary logistic regression model (0 = HE; 1 = SE). The level of significance was set at *p* < 0.05. For visualization of the results, GraphPad Prism version 7.00 for Windows (GraphPad Software, La Jolla, CA, USA) was used.

## 3. Results

The 122 enrolled cows comprised 91 (74.6%) multiparous and 31 (25.4%) primiparous cows. VDS 0, 1, 2, and 3 were found in 43.4% (*n* = 53), 19.7% (*n* = 24), 18.9% (*n* = 23), and 18.0% (*n* = 22) of the animals, respectively. Thus, the prevalence of CE (VDS 1, 2, or 3) was 56.6%. The prevalence of SE (VDS 0 and ≥5% PMN) was 30.3% (*n* = 37), and 13.1% (*n* = 16) of the cows were HE cows (VDS 0 and <5% PMN). There was no significant difference in the prevalence of CE and SE between primi- and multiparous animals. 

### 3.1. Composition and Dynamics of Cultivable Anaerobic Intrauterine Microbiota

**Figure 1:**
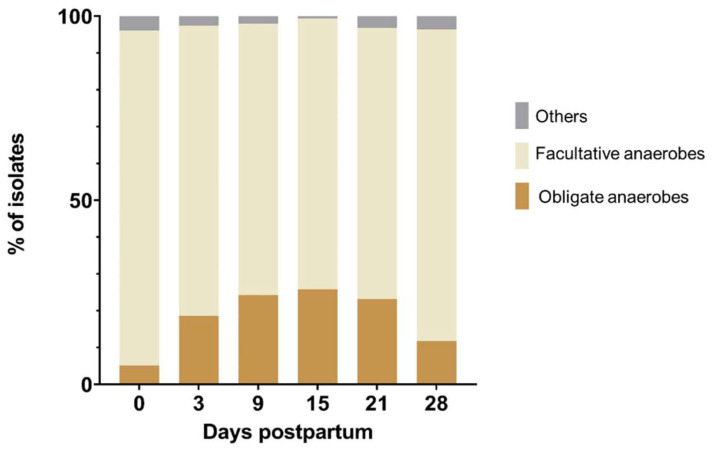
Relative abundance of obligate anaerobes, facultative anaerobes, and other bacteria (microaerophiles and probable new species without known metabolism) at the day of calving (day 0) and on days 3, 9, 15, 21, and 28 postpartum.

A total of 1858 isolates were recovered, of which 18.6% were obligate anaerobes, and 78.8% were facultative anaerobes, while bacteria with other types of metabolism, such as microaerophilic bacteria, comprised only a small fraction (2.6%). The prevalence of the obligate and facultative anaerobes varied between the different sampling days, as shown in Figure 1. The relative abundance of obligate anaerobes increased until day 15 and decreased thereafter, whereas facultative anaerobes showed the opposite progression of infection.

**Figure 2:**
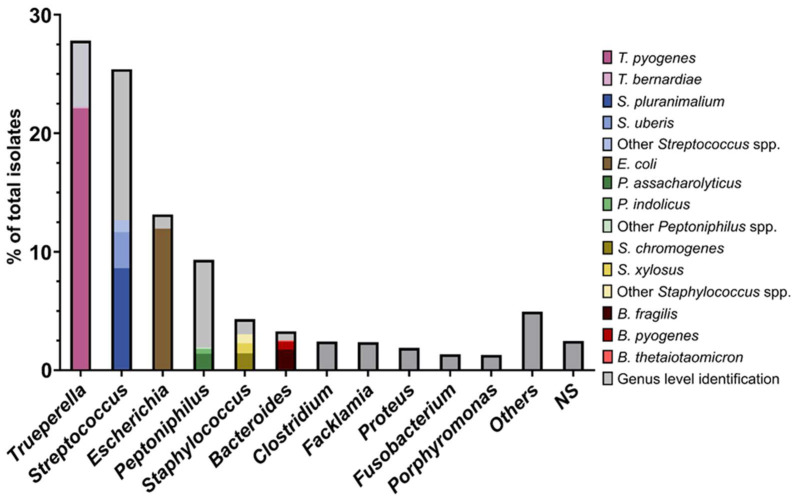
Composition of the anaerobic cultivable microbiota found in the postpartum bovine uterus during the first 28 days postpartum. Relative abundance was calculated for genera with prevalence higher than 1% (*n* = 1858 isolates). In addition, relative abundances are shown on species level for the six most frequently isolated genera. Genera representing less than 1% of the total number of isolates were combined as others. NS (potentially new species): bacteria showing <98.65% 16S rRNA similarity to the next phylogenetic relative species and may potentially represent a new taxa.

The intrauterine anaerobic cultivable microbiota showed high diversity, including 36 different genera. The most abundant bacterial genera found in the postpartum uterus were *Trueperella* (27.8%; 517/1858), *Streptococcus* (25.4%; 472/1858), *Escherichia* (13.1%; 244/1858), and *Peptoniphilus* (9.3%; 173/1858), as depicted in Figure 2. The most common species found were *T. pyogenes* (22.2%; 413/1858)*, E. coli* (12.1%; 225/1858), and *Streptococcus pluranimalium* (8.6%; 160/1858). *Streptococcus* was the genus with the greatest intra-genera diversity, with 14 different isolated species. The most common representative of the genus *Streptococcus* was *S. pluranimalium*, with 34.0% (160/472) of the total *Streptococcus* isolates belonging to this species (Figure 2). 

From the genus *Staphylococcus,* 10 different species were isolated, mainly represented by *Staphylococcus chromogenes* (33.8%, 27/80), and the genus *Clostridium* was represented by six different species, with *Clostridium perfringens* as the most abundant representative (48.9%, 22/45). By contrast, the genera *Trueperella* and *Escherichia* showed low species diversity, being predominantly represented by *T. pyogenes* and *E. coli*. Various genera had a prevalence < 1%, such as *Enterococcus* (0.97%), *Bacillus* (0.8%), *Corynebacterium* (0.4%), and *Prevotella* (0.3%). The majority of isolates, i.e., 80.3% (139/173), belonging to the genus *Peptoniphilus* could only be identified on the genus level. Only three different *Peptoniphilus* species were identifiable on a species level, with *Peptoniphilus asaccharolyticus* as the main representative species (14.5%, 25/173). 

**Figure 3:**
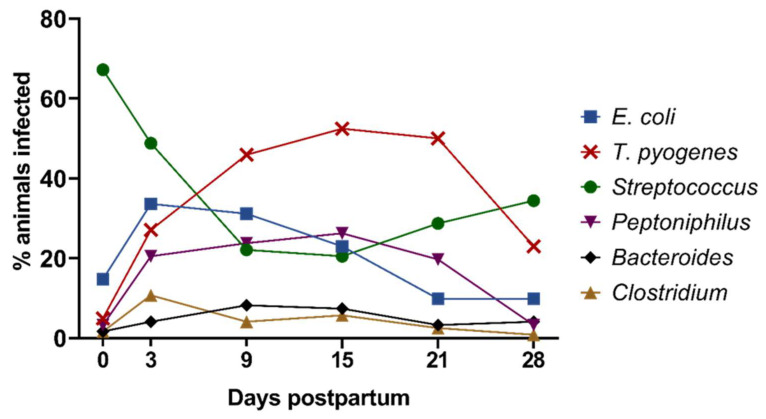
Percentage of animals positive for *E. coli*, *T. pyogenes*, *Streptococcus* spp., *Peptoniphilus* spp., *Bacteroides* spp., and *Clostridium* spp. at the day of calving (day 0) and on days 3, 9, 15, 21, and 28 postpartum.

During the first 28 days pp, compositional changes in the cultivable anaerobic microbiota were observed (Figure 3). At the day of calving (day 0), *Streptococcus* spp. dominated the uterine microbiota and was found in more than two-thirds of the animals (67.2%; 82/122). From day 3 onwards, *Streptococcus* spp. rapidly declined until day 15 (20.5%; 25/122) and slightly increased again until day 28 pp. *T. pyogenes* followed an opposite infection pattern and was rarely recovered on day 0, reached its maximum on day 15 with 52.5% positive animals (64/122), and declined thereafter. *Peptoniphilus* spp. showed a similar trend as *T. pyogenes*, with 23.8% (29/122) and 26.2% (32/122) positive animals on days 9 and 15 pp, respectively. *E. coli* and *Clostridium* spp. peaked on day 3 pp (33.6%; 41/122 and 10.7%; 13/122), followed by lower detection rates thereafter. *Bacteroides* spp. showed a flat infection curve and was most frequently detected on day 9 pp (8.2%; 10/122). 

### 3.2. Co-Occurrence of Different Bacterial Species and Genera

It was striking that early *Escherichia* infections (day 0, 3, and day 9 pp) correlated with the later appearance of *Trueperella* (day 15, 21, and day 28 pp; *p*-values ranging from 0.003 to 0.047, Figure S1). On the same sampling days, *Escherichia* correlated with *Bacteroides* and *Clostridium* (day 3 pp; *p* = 0.029 and *p* = 0.030, respectively) and with the presence of *Streptococcus* and *Peptoniphilus* spp. (day 9 pp; *p* = 0.049 and *p* = 0.025, respectively). On the species level, the presence of *E. coli* on day 0 pp correlated with *Peptoniphilus indolicus* (*p* = 0.016) and *S. pluranimalium* (*p* = 0.041) on the same sampling day. The presence of *E. coli* and *P. indolicus* tended to correlate (*p* = 0.067) on day 15 and with *F. necrophorum* on day 28 pp (*p* = 0.055). 

The presence of *T. pyogenes* correlated with *Peptoniphilus* spp. on day 0 and day 3 pp (both *p* < 0.001) and day 21 pp (*p* = 0.001), while the presence of *T. pyogenes* correlated also with the presence of the obligate anaerobe *Porphyromonas levii* on day 3 (*p* = 0.004). 

Furthermore, correlations between *Streptococcus pluraniumalium* and *Staphylococcus* spp. observed were on day 0 (*p* = 0.046) and day 28 pp (*p* = 0.052), respectively. On the genus level, different anaerobes such as *Porphyromonas* and *Bacteroides* were isolated simultaneously with other anaerobes or *Trueperella* and showed a significant co-occurrence in the sampled cows, as shown in the heat map Supplementary Figure S1. 

### 3.3. Prevalence of Bacteriological Findings in HE, CE, and SE Cows

**Figure 4:**
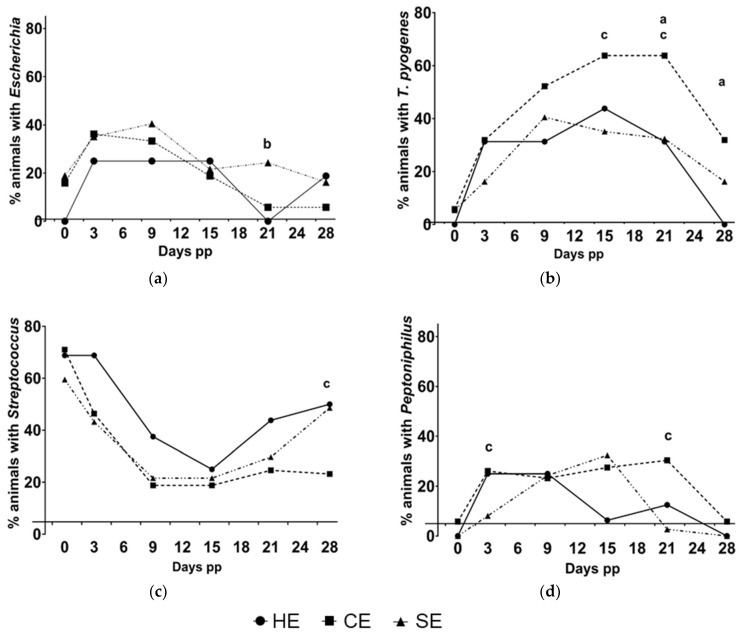
Percentage of healthy cows (HE, *n* = 16) and cows with clinical (CE, *n* = 69) and subclinical (SE, *n* = 37) endometritis positive for (**a**) *Escherichia*, (**b**) *T. pyogenes*, (**c**) *Streptococcus* spp., and (**d**) *Peptoniphilus* spp. at the day of calving (day 0) and on days 3, 9, 15, 21, and 28 postpartum. a, significant difference between CE and healthy animals; b, significant difference between SE and healthy animals; c, significant difference between SE and CE animals; *p* < 0.05, respectively.

An overview on the prevalence of bacteriologically positive animals in HE, CE, and SE cows for the most abundant genera is provided in Figure 4. Certain bacteria were predominantly found in CE and SE animals or in HE cows on specific days.

*T. pyogenes* was recovered more frequently from animals with CE than from HE animals on day 21 (63.8%, 44/69 vs. 31.3%, 5/16; *p* = 0.018) and on day 28 pp (31.9%, 22/69 vs. 0.0%, 0/16; *p* = 0.009). *T. pyogenes* was also found more frequently in CE than in SE on day 15 (63.8%, 44/69 vs. 35.1%, 13/37; *p* = 0.018) and 21 pp (63.8%, 44/69 vs. 32.4%, 12/37; *p* = 0.002). *Escherichia* was found more frequently on day 21 pp in animals with SE than in HE animals (21.6%, 8/37 vs. 0.0%, 0/16; *p* = 0.044) and in CE cows (21.6%, 8/37 vs. 5.8%,4/69; *p* = 0.023). Additionally, the prevalence of the genus *Streptococcus* spp. on day 28 pp tended to be greater in HE animals than in cows with CE (50.0%, 8/16 vs. 23.2%, 16/69; *p* = 0.060) and was more frequently found in SE than in CE cows (48.6%, 18/37 vs. 23.2%, 16/69; *p* = 0.009). The species *S. pluranimalium* was found more frequently in HE animals than in CE cows on day 28 pp (37.5%, 6/16 vs. 7.2%, 5/69; *p* = 0.005). *Peptoniphilus* spp. was more frequently identified in CE than in SE cows on days 3 (26.1%, 18/69 vs. 8.1%, 3/37; *p* = 0.027) and 21 pp (30.4%, 21/69 vs. 2.7%, 1/37; *p* < 0.001). Furthermore, some genera were found exclusively in HE or in endometritic animals. *Fusobacterium* and *Bacteroides* were exclusively in endometritic animals (with SE or CE) throughout the entire sampling period but not observed in HE animals. Several genera with prevalence < 1% were identified exclusively in HE, CE, or SE animals. *Peptostreptococcus* and *Enterobacter* were exclusively recovered from animals with CE. On the contrary, *Lactobacillus* spp. were found exclusively in animals with SE, and *Gallibacterium* spp. were found exclusively in HE animals.

### 3.4. Prevalence of Bacteriological Findings in Primi- and Multiparous Cows

**Figure 5:**
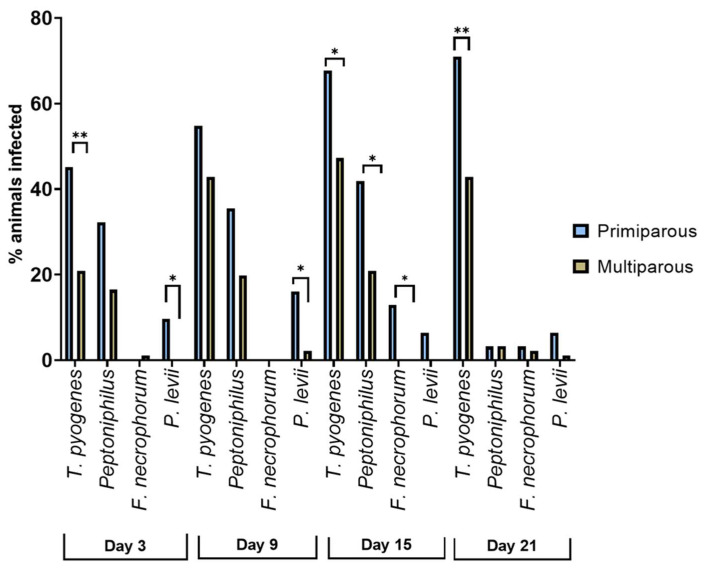
Differences in the prevalence of *T. pyogenes*, *Peptoniphilus* spp., *F. necrophorum*, and *P. levii* in primiparous (*n* = 31) vs. multiparous cows (*n* = 91). * indicates 0.01< *p* < 0.05; ** indicates *p* < 0.01.

The prevalence of species differed significantly between primi- and multiparous animals (see Figure 5). *T. pyogenes* was more prevalent in primiparous than in multiparous cows on day 3 (45.2%, 14/31 vs. 20.9%, 19/91; *p* = 0.009), day 15 (67.7%, 21/31 vs. 47.3%, 43/91; *p* = 0.049), and day 21 pp (71.0%, 22/31 vs. 42.9%, 39/91; *p* = 0.007). The anaerobic *Peptoniphilus* spp. were also more frequently identified in samples from primiparous cows on day 15 pp (41.9%, 13/31 vs. 20.9%, 19/91; *p* = 0.021) than in multiparous cows. In addition, *F. necrophorum* was recovered exclusively from primiparous cows on day 15 pp (12.9%, 4/31; *p* = 0.004). *Porphyromonas levii* was found more frequently in primiparous than in multiparous cows on day 3 (9.7%, 3/31 vs. 0.0%, 0/91; *p* = 0.015) and day 9 (16.1%, 5/31 vs. 2.2%, 2/91; *p* = 0.011). 

### 3.5. Dynamics of the Microbiota in Relation to the Uterine Hhealth Status

**Figure 6:**
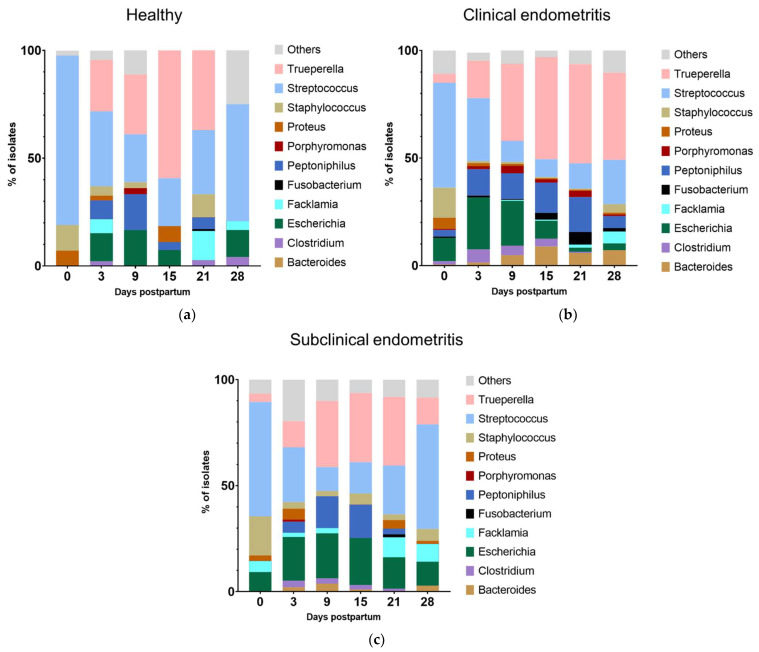
Relative abundance of uterine microbiota from isolates (*n* = 1858) collected at the day of calving (day 0) and on days 3, 9, 15, 21, and 28 postpartum in (**a**) healthy cows (*n* = 16), (**b**) cows with clinical endometritis (*n* = 69), and cows with (**c**) subclinical endometritis (*n* = 37).

Shifts in the composition of microbiota were observed throughout the postpartum period in HE, CE, and SE cows. The relative abundance of certain bacteria was linked to the uterine health status of the cows (Figure 6). 

Notably, *Trueperella* was completely absent in HE cows on the day of calving (day 0) but already present in CE and SE cows. On day 15, the relative abundance of *Trueperella* was significantly greater in HE (59.3%) cows than SE cows (32.6%, *p* = 0.012). Nevertheless, *Trueperella* was eliminated by day 28 in HE animals, while it was still present in CE and SE cows, with a greater abundance in CE than in SE (46.1% vs. 32.4%, *p* = 0.041). 

Similar to *Trueperella*, *Escherichia* was absent in HE animals on the day of calving while present in CE and SE cows. In both groups, *Escherichia* abundance was significantly greater than in HE (CE: 10.9%, *p* = 0.018 and SE: 9.2%, *p* = 0.050). On day 21 pp, the relative abundance of *Escherichia* was greater in SE (14.9%) that in HE (0.0%, *p* = 0.015) or in CE (2.0%, *p* < 0.001) cows. On day 28 pp, *Escherichia* was found in greater abundances in SE (11.3%) than in CE (3.2%, *p* = 0.030) cows. 

The genera *Streptococcus* and *Staphylococcus*, *Proteus*, and *Facklamia* were positively related to uterine health. On the day of calving, the majority of isolates from HE belonged to the genus *Streptococcus* (78.6%), while it was less abundant in CE (48.7%, *p* < 0.001) or SE (53.9%, *p* = 0.008) cows. *Streptococcus* was also more abundant in HE animals compared to CE cows on day 9 (HE: 22.2% vs. CE: 9.7%, *p* = 0.029), day 15 (HE: 22.2% vs. CE: 8.3%, *p* = 0.037), day 21 (HE: 29.7% vs. CE: 11.8%, *p* = 0.004), and on day 28 pp (HE: 54.2% vs. CE: 20.6%, *p* = 0.001). On day 15 pp, *Proteus* was more abundant in HE animals (HE: 10.8%) than in CE (0.5%, *p* = 0.002) or SE cows (0.0%, *p* = 0.047). HE animals showed also higher abundances for *Staphylococcus* (HE: 10.8% vs. CE: 0.5%, *p* = 0.002) and *Facklamia* spp. (HE: 13.5% vs. CE: 1.5%, *p* = 0.003) on day 21 pp than CE animals. In addition, *Facklamia* spp. was more abundant in HE than in CE cows on day 3 pp (HE: 6.5% vs. CE: 0.9%, *p* = 0.041). 

*Bacteroides* spp. appeared more frequently in CE than in SE animals on day 15 pp (CE: 8.9% vs. SE: 1.1%, *p* = 0.010) and day 21 pp (CE: 5.9% vs. SE: 0.0%, *p* = 0.040). Other obligate anaerobic bacteria such as *Bacteroides, Clostridium*, and *Fusobacterium* were found mainly in endometritic cows rather than healthy cows but with low abundances and no significant differences between groups. 

### 3.6. Relative Risk for Different Vaginal Discharge Scores and Odds Ratio for the Occurrence Subclinical Endometritis

Moreover, by using a multinomial logistic regression model, the relative risk for the occurrence of abnormal vaginal discharge (with VDS 0 as reference) was calculated for the presence of certain bacteria on days 0, 3, 9, 15, 21, and 28 pp. It was observed that the occurrence of *Streptococcus* spp. on day 15 pp was related to an increased risk for VDS 3 (RRR = 4.817, CI = 1.23–18.87, *p* = 0.024). Additionally, *Peptoniphilus* spp. correlated with an increased risk on day 21 for VDS 3 (RRR = 12.44, CI = 2.34–66.10, *p* = 0.003). Furthermore, the presence of *T. pyogenes* on day 21 pp showed an increased relative risk for VDS 2 (RRR = 4.13, CI = 1.37–12.44, *p* = 0.012) and VDS 3 (RRR = 5.08, CI = 1.34–19.23, *p* = 0.017), as shown in Table 1. However, the binary logistic regression did not reveal a correlation between the bacterial findings and the presence of SE. 

## 4. Discussion

Postpartum uterine microbiota and the development of uterine diseases such as CE and SE in dairy cows are highly dynamic, interlinked processes, but studies on the characterization of the microbiota composition during disease development are still limited [13,17,18]. Especially, the role of facultative and obligate anaerobes for the pathogenesis of CE and SE is far from understood. By using a sequential approach including six sampling time points during the first 28 days postpartum, this study aimed at examining health-status-related fluctuations within the anaerobic cultivated microbiota during disease development. 

About one-fifth of the isolates retrieved from the uterine and grown under anaerobic conditions were obligate anaerobic bacteria, which showed a dynamic infection pattern throughout the postpartum period. The predominant obligate genera were *Peptoniphilus*, *Bacteroides*, *Clostridium*, and *Fusobacterium*. At the day of calving, the facultative anaerobes clearly dominated the intrauterine microbiota, but until day 15 postpartum, they were partly replaced by obligate anaerobes. It could be speculated that the growth conditions improved for obligate anaerobes over time after calving, when the cervix is closed. On day 28 postpartum, however, the relative abundance of obligate anaerobes decreased again. Therefore, we assume that the fraction of obligate anaerobes in our study did not belong to the physiological microbiota found in virgin or pregnant cows [2,3] but are presumably involved in the pathogenesis of uterine diseases. Indeed, the obligate anaerobe *Peptoniphilus* spp. was associated with an increased risk for the development of purulent vaginal discharge. This is supported by studies showing that *Peptoniphilus* spp. were strongly associated with CE [26,27]. 

The facultative anaerobes *T. pyogenes, Streptococcus* spp., and *E. coli* were the most abundant bacteria in the present study, which parallels the findings after aerobic cultivation [13,28]. However, the obligate anaerobes could be underrepresented in our culture-dependent study compared to culture-independent approaches because of their fastidious growth. On the other hand, Santos and Bicalho [17] did not find *T. pyogenes* and *E. coli* in high abundances in cows with CE and concluded that culture-dependent studies overestimate these bacterial species. The consistent finding that *T. pyogenes* increased the risk for purulent vaginal discharge and its strong association to CE is supported by other studies [13,16,18]. Therefore, we propose to interpret results from culture-dependent and -independent studies as complementary findings contributing to the same overall picture, as suggested by Sicsic et al. [6]. 

Our analyses on the compositional changes in the uterine microbiota revealed that *T. pyogenes* was more abundant in healthy animals than in CE or SE cows on day 15 pp. In HE animals, however, *T. pyogenes* was completely eliminated until day 28 pp, while it was still present in CE and SE cows. This finding enhances our understanding of the development of uterine diseases, as it supports the concept that disease development depends on the ability of the animal to tolerate and eliminate pathogens [29]. 

*T. pyogenes* is a well-defined potential pathogen for bovine endometritis [1], and comprehensive information is available about its pathogenicity [30,31]. However, there is still a lack of information about the involvement of other bacteria in the establishment of uterine *Trueperella* infections, as mentioned recently [18]. In our study, the co-occurrence of *T. pyogenes* and *Peptoniphilus* spp. during the postpartum period and co-occurrence of *F. necrophorum* and *Bacteroides* spp. with *T. pyogenes* at the later postpartum period was observed. This further strengthens previous findings indicating that *T. pyogenes* acts synergistically with obligate anaerobes [32], which could be of high clinical relevance. Further research is still needed to elucidate the pathological mechanisms behind these interactions. The strong correlation between *T. pyogenes* and *Peptoniphilus* spp. throughout the entire sampling period confirms previous studies where *T. pyogenes* and *Peptoniphilus* spp. correlated on day 35 pp [18] and justifies experimental in vitro studies on the pathogenicity of *Peptoniphilus* spp. 

Notably, *Streptococcus* spp. and *T. pyogenes* showed an inverse proportional prevalence. Previous studies indicated that *Streptococcus* spp. are abundant during the first week postpartum and that the peak of *T. pyogenes* abundance is on day 15 postpartum [13,33]. This was also seen in our study, with a huge proportion of cows being positive for *Streptococcus* spp. on day 0 and day 28 pp, while the prevalence of *T. pyogenes* peaked on day 15 but was lower on day 0 and 28 pp. These results may indicate a dysbiotic relationship between *Streptococcus* spp. and *T. pyogenes*.

*S. pluranimalium* represented more than one-third of the total *Streptococcus* isolates, and *S. uberis* was the second most frequent *Streptococcus* found. While there is increasing evidence for the contribution of *S. uberis* to uterine diseases [19,34], less is known about intrauterine *S. pluranimalium* infections despite its high abundance. In the current study, *S. pluranimalium* was less prevalent in CE than in HE cows, suggesting a beneficial effect for uterine health. On the other hand, we found a co-prevalence with the pathogen *E. coli* on the genera level, and *Streptococcus spp*. increased the risk for purulent vaginal discharge. *S. pluranimalium* has also been isolated from abortion material [35] but was frequently detected at the day of insemination without any effect on uterine health and fertility. Therefore, its contribution to uterine health or disease is still in need of clarification, but it is tempting to speculate that some members of the genus *Streptococcus* act as opportunistic pathogens, such as *Streptococcus uberis* [19]. 

The presence of *Peptostreptococcus* spp. only in endometritic animals is in accordance with findings from previous studies [36,37]. The discovery of *Lactobacillus* spp. only in SE but not in CE animals fortifies previous studies showing that *Lactobacilli* can be found in animals with no clinical signs of uterine diseases [38] or are enriched in SE cows [27]. Furthermore, there is some evidence that intrauterine infusion of *Lactobacilli* might improve the fertility of cows with SE and healthy animals [39]. The analysis of fertility data, however, was beyond the scope of our study. Further studies are needed to elucidate the effect of certain bacteria with potential health benefits, such as *Lactobacilli* species, on reproductive performance. 

On the day of diagnosis, *Escherichia* was detected more frequently in animals with SE than in healthy animals, which is in accordance with previous results [27]. In the binary logistic regression, however, bacterial findings were not related to SE, indicating that bacterial infections play a minor role for SE development, as previously described [9,12]. 

Additionally, the parity of the animals had an effect on the prevalence of some bacterial species. It was striking that primiparous cows showed greater prevalence of bacteria than multiparous cows and that bacteria with significant differences included bacteria generally considered as pathogens, such as *Fusobacterium* spp., *P. levii*, and *T. pyogenes* [1,40]. Primiparous cows have a less-trained immune system, which allows the assumption that they are also less resilient to uterine bacterial infections. The fact that the observed parity-effect was already present on day 3, 9, and 15 and the finding that parity had no effect on the development of CE or SE leads to the assumption that primiparous cows manage to develop a competent immune system during the early postpartum period, which successfully limits the growth of pathogens. 

The panel of isolates retrieved from the uterine of cows with distinctive health builds the basis for further experimental studies on the pathogenicity of specific bacterial groups. Nevertheless, there are limitations of the study that can be attributed to the sampling technique, which may have an effect on the detected species [33], or the use of a culture-based approach. In addition, the study was performed only on one single farm. These limitations can be overcome by future studies with larger animal population and the use of multiple sampling and analysis techniques (uterine lavage and/or uterine swabs and simultaneous analysis with a culture-dependent and culture-independent approach). Such studies will lead to further clarification of the role of the anaerobic and aerobic bacteria and their interactions with regard to the development of bovine endometritis.

## 5. Conclusions

In summary, our study provides new information on the dynamics of the cultivable anaerobic part of the postpartum uterine microbiome in dairy cows before, during, and after disease diagnosis. Facultative anaerobes such as *T. pyogenes*, *E. coli*, and *Streptococcus* spp. and obligate anaerobes such as *Peptoniphilus*, *Bacteroides*, *Fusobacterium*, and *Clostridium* spp. represented a major part of the cultivable microbiota. Remarkable shifts were observed in the composition of microbiota throughout the postpartum period. *T. pyogenes* and *Peptoniphilus* spp. on day 21 pp increased the risk for purulent vaginal discharge, and their early co-occurrence indicated bacterial synergisms presumably decisive for CE development. Representative isolates *T. pyogenes* and *Peptoniphilus* spp. retrieved from the bovine uterine in the frame of this study might provide a suitable basis for future co-cultivation studies to elucidate potential synergistic interactions between the two species. The inverse isolation rates between *S. pluranimalium* and *T. pyogenes* might indicate a competition with a potentially beneficial effect for uterine health, which warrants further investigation. 

## Figures and Tables

**Table 1:** Relative risk ratio and confidence intervals for vaginal discharge score (VDS) 1, 2, and 3 (in comparison to VDS 0) as assessed on day 21 postpartum in relation to the bacterial findings of *T. pyogenes*, *E. coli*, *Streptococcus* spp., and *Peptoniphilus* spp. on days 0, 3, 9, 15, and 21 postpartum as calculated by multinomial logistic regression. Only days with significant results are shown.

Bacterial Finding	VDS 1		VDS 2		VDS 3	
	RRR	95% CI	RRR	95% CI	RRR	95% CI
*Streptococcus*, day 15	0.33	0.67–1.66	0.15	0.17–1.29	4.82 *	1.23–18.87
*Peptoniphilus*, day 21	2.25	0.44–11.67	3.14	0.66–14.95	12.44 **	2.34–66.10
*T. pyogenes*, day 21	1.64	0.58–4.65	4.13 *	1.37–12.44	5.08 **	1.34–19.23

RRR, relative risk ratio; CI, confidence interval; * *p*-value < 0.05; ** *p*-value < 0.01.

## Data Availability

Not applicable.

## Supplementary Materials

The following supporting information can be downloaded at: https://www.mdpi.com/article/10.3390/ani13010082/s1: Figure S1: Heatmap of *p*-values showing the co-occurrence of different bacterial findings at each given day postpartum at genus level.

## References

[1] Carneiro L.C., Cronin J.G., Sheldon I.M. (2016). Mechanisms linking bacterial infections of the bovine endometrium to disease and infertility. Reprod. Biol..

[2] Moore S.G., Ericsson A.C., Poock S.E., Melendez P., Lucy M.C. (2017). Hot topic: 16S rRNA gene sequencing reveals the microbiome of the virgin and pregnant bovine uterus. J. Dairy Sci..

[3] Karstrup C.C., Klitgaard K., Jensen T.K., Agerholm J.S., Pedersen H.G. (2017). Presence of bacteria in the endometrium and placentomes of pregnant cows. Theriogenology.

[4] Ballas P., Reinländer U., Schlegl R., Ehling-Schulz M., Drillich M., Wagener K. (2021). Characterization of intrauterine cultivable aerobic microbiota at the time of insemination in dairy cows with and without mild endometritis. Theriogenology.

[5] Paiano R.B., Moreno L.Z., Gomes V.T.M., Parra B.M., Barbosa M.R., Sato M.I.Z., Bonilla J., Pugliesi G., Baruselli P.S., Moreno A.M. (2022). Assessment of the main pathogens associated with clinical and subclinical endometritis in cows by culture and MALDI-TOF mass spectrometry identification. J. Dairy Sci..

[6] Sicsic R., Goshen T., Dutta R., Kedem-Vaanunu N., Kaplan-Shabtai V., Pasternak Z., Gottlieb Y., Shpigel N.Y., Raz T. (2018). Microbial communities and inflammatory response in the endometrium differ between normal and metritic dairy cows at 5–10 days post-partum. Vet. Res..

[7] Bicalho M.L.S., Machado V.S., Higgins C.H., Lima F.S., Bicalho R.C. (2017). Genetic and functional analysis of the bovine uterine microbiota. Part I: Metritis versus healthy cows. J. Dairy Sci..

[8] Bicalho M.L.S., Lima S., Higgins C.H., Machado V.S., Lima F.S., Bicalho R.C. (2017). Genetic and functional analysis of the bovine uterine microbiota. Part II: Purulent vaginal discharge versus healthy cows. J. Dairy Sci..

[9] Madoz L.V., Giuliodori M.J., Migliorisi A.L., Jaureguiberry M., de la Sota R.L. (2014). Endometrial cytology, biopsy, and bacteriology for the diagnosis of subclinical endometritis in grazing dairy cows. J. Dairy Sci..

[10] Prunner I., Wagener K., Pothmann H., Ehling-Schulz M., Drillich M. (2014). Risk factors for uterine diseases on small- and medium-sized dairy farms determined by clinical, bacteriological, and cytological examinations. Theriogenology.

[11] Baranski W., Podhalicz-Dziegielewska M., Zdunczyk S., Janowski T. (2012). The diagnosis and prevalence of subclinical endometritis in cows evaluated by different cytologic thresholds. Theriogenology.

[12] Prunner I., Pothmann H., Wagener K., Giuliodori M., Huber J., Ehling-Schulz M., Drillich M. (2014). Dynamics of bacteriologic and cytologic changes in the uterus of postpartum dairy cows. Theriogenology.

[13] Wagener K., Prunner I., Pothmann H., Drillich M., Ehling-Schulz M. (2015). Diversity and health status specific fluctuations of intrauterine microbial communities in postpartum dairy cows. Vet. Microbiol..

[14] Miranda-CasoLuengo R., Lu J., Williams E.J., Miranda-CasoLuengo A.A., Carrington S.D., Evans A.C.O., Meijer W.G. (2019). Delayed differentiation of vaginal and uterine microbiomes in dairy cows developing postpartum endometritis. PLoS ONE.

[15] Sens A., Heuwieser W. (2013). Presence of Escherichia coli, Trueperella pyogenes, alpha-hemolytic streptococci, and coagulase-negative staphylococci and prevalence of subclinical endometritis. J. Dairy Sci..

[16] Werner A., Suthar V., Plöntzke J., Heuwieser W. (2012). Relationship between bacteriological findings in the second and fourth weeks postpartum and uterine infection in dairy cows considering bacteriological results. J. Dairy Sci..

[17] Santos T.M., Bicalho R.C. (2012). Diversity and succession of bacterial communities in the uterine fluid of postpartum metritic, endometritic and healthy dairy cows. PLoS ONE.

[18] Pascottini O.B., Van Schyndel S.J., Spricigo J.F.W., Rousseau J., Weese J.S., LeBlanc S.J. (2020). Dynamics of uterine microbiota in postpartum dairy cows with clinical or subclinical endometritis. Sci. Rep..

[19] Ballas P., Gabler C., Wagener K., Drillich M., Ehling-Schulz M. (2020). *Streptococcus uberis* strains originating from bovine uteri provoke upregulation of pro-inflammatory factors mRNA expression of endometrial epithelial cells in vitro. Vet. Microbiol..

[20] Danesh Mesgaran S., Gärtner M.A., Wagener K., Drillich M., Ehling-Schulz M., Einspanier R., Gabler C. (2018). Different inflammatory responses of bovine oviductal epithelial cells in vitro to bacterial species with distinct pathogenicity characteristics and passage number. Theriogenology.

[21] Sheldon I.M., Lewis G.S., LeBlanc S., Gilbert R.O. (2006). Defining postpartum uterine disease in cattle. Theriogenology.

[22] Westermann S., Drillich M., Kaufmann T.B., Madoz L.V., Heuwieser W. (2010). A clinical approach to determine false positive findings of clinical endometritis by vaginoscopy by the use of uterine bacteriology and cytology in dairy cows. Theriogenology.

[23] Kasimanickam R., Duffield T.F., Foster R.A., Gartley C.J., Leslie K.E., Walton J.S., Johnson W.H. (2005). A comparison of the cytobrush and uterine lavage techniques to evaluate endometrial cytology in clinically normal postpartum dairy cows. Can. Vet. J..

[24] Ballas P., Rückert C., Wagener K., Drillich M., Kämpfer P., Busse H.J., Ehling-Schulz M. (2020). *Corynebacterium endometrii* sp. Nov., isolated from the uterus of a cow with endometritis. Int. J. Syst. Evol. Microbiol..

[25] Kim M., Oh H.S., Park S.C., Chun J. (2014). Towards a taxonomic coherence between average nucleotide identity and 16S rRNA gene sequence similarity for species demarcation of prokaryotes. Int. J. Syst. Evol. Microbiol..

[26] Machado V.S., Oikonomou G., Bicalho M.L.S., Knauer W.A., Gilbert R., Bicalho R.C. (2012). Investigation of postpartum dairy cows’ uterine microbial diversity using metagenomic pyrosequencing of the 16S rRNA gene. Vet. Microbiol..

[27] Wang M.L., Liu M.C., Xu J., An L.G., Wang J.F., Zhu Y.H. (2018). Uterine microbiota of dairy cows with clinical and subclinical endometritis. Front. Microbiol..

[28] Wagener K., Grunert T., Prunner I., Ehling-Schulz M., Drillich M. (2014). Dynamics of uterine infections with *Escherichia coli*, *Streptococcus uberis* and *Trueperella pyogenes* in post-partum dairy cows and their association with clinical endometritis. Vet. J..

[29] Sheldon I.M., Molinari P.C.C., Ormsby T.J.R., Bromfield J.J. (2020). Preventing postpartum uterine disease in dairy cattle depends on avoiding, tolerating and resisting pathogenic bacteria. Theriogenology.

[30] Ibrahim M., Peter S., Wagener K., Drillich M., Ehling-Schulz M., Einspanier R., Gabler C. (2017). Bovine endometrial epithelial cells scale their pro-inflammatory response *in vitro* to pathogenic *Trueperella pyogenes* isolated from the bovine uterus in a strain-specific manner. Front. Cell. Infect. Microbiol..

[31] Piersanti R.L., Zimpel R., Molinari P.C.C., Dickson M.J., Ma Z., Jeong K.C., Santos J.E.P., Sheldon I.M., Bromfield J.J. (2019). A model of clinical endometritis in Holstein heifers using pathogenic *Escherichia coli* and *Trueperella pyogenes*. J. Dairy Sci..

[32] Bicalho M.L.S., Machado V.S., Oikonomou G., Gilbert R.O., Bicalho R.C. (2012). Association between virulence factors of *Escherichia coli*, *Fusobacterium necrophorum*, and *Arcanobacterium pyogenes* and uterine diseases of dairy cows. Vet. Microbiol..

[33] Knudsen L.R., Karstrup C.C., Pedersen H.G., Angen O., Agerholm J.S., Rasmussen E.L., Jensen T.K., Klitgaard K. (2016). An investigation of the microbiota in uterine flush samples and endometrial biopsies from dairy cows during the first 7 weeks postpartum. Theriogenology.

[34] Ledgard A.M., Smolenski G.A., Henderson H., Lee R.S.F. (2015). Influence of pathogenic bacteria species present in the postpartum bovine uterus on proteome profiles. Reprod. Fertil. Dev..

[35] Foster G., Barley J., Howie F., Falsen E., Moore E., Twomey D.F., Wragg P., Whatmore A.M., Stubberfield E. (2008). *Streptococcus pluranimalium* in bovine reproductive disease. Vet. Rec..

[36] Peng Y., Wang Y., Hang S., Zhu W. (2013). Microbial diversity in uterus of healthy and metritic postpartum Holstein dairy cows. Folia Microbiol..

[37] Jeon S.J., Galvão K.N. (2018). An advanced understanding of uterine microbial ecology associated with metritis in dairy cows. Genom. Inform..

[38] Gärtner M.A., Bondzio A., Braun N., Jung M., Einspanier R., Gabler C. (2015). Detection and characterisation of *Lactobacillus* spp. in the bovine uterus and their influence on bovine endometrial epithelial cells in vitro. PLoS ONE.

[39] Peter S., Gärtner M.A., Michel G., Ibrahim M., Klopfleisch R., Lübke-Becker A., Jung M., Einspanier R., Gabler C. (2018). Influence of intrauterine administration of *Lactobacillus buchneri* on reproductive performance and pro-inflammatory endometrial mRNA expression of cows with subclinical endometritis. Sci. Rep..

[40] Galvão K.N., Bicalho R.C., Jeon S.J. (2019). Symposium review: The uterine microbiome associated with the development of uterine disease in dairy cows. J. Dairy Sci..

