# Supplementary figures and images for: Dynamics and Diversity of Intrauterine Anaerobic Microbiota in Dairy Cows with Clinical and Subclinical Endometritis
Source: Animals (Basel). 2022 Dec 26;13(1):82. doi: 10.3390/ani13010082 (PMC9817838; doi:10.3390/ani13010082)

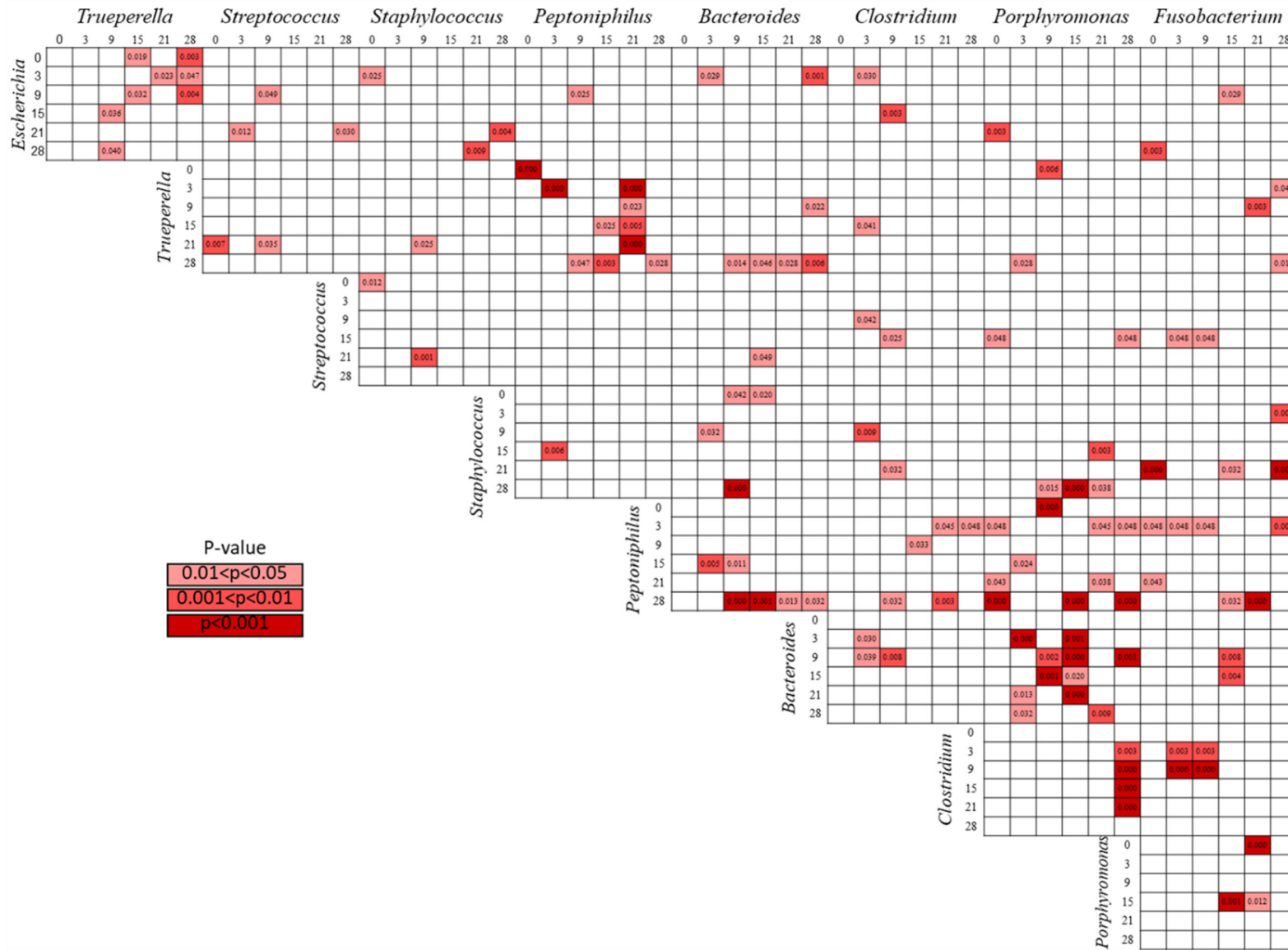

Supplement: Supplementary file 1 [file animals-13-00082-s001.zip › animals-2120063-supplementary.pdf]
